# Molecular Dynamics Investigation of MFS Efflux Pump MdfA Reveals an Intermediate State between Its Inward and Outward Conformations

**DOI:** 10.3390/ijms24010356

**Published:** 2022-12-26

**Authors:** Ying Li, Xizhen Ge

**Affiliations:** College of Biochemical Engineering, Beijing Union University, Beijing 100023, China

**Keywords:** major facilitator superfamily, efflux pump, conformational transition, intermediate state, molecular dynamics

## Abstract

Multidrug resistance poses a major challenge to antibiotic therapy. A principal cause of antibiotic resistance is through active export by efflux pumps embedded in the bacterial membrane. Major facilitator superfamily (MFS) efflux pumps constitute a major group of transporters, which are often related to quinolone resistance in clinical settings. Although a rocker-switch model is proposed for description of their conformational transitions, detailed changes in this process remain poorly understood. Here we used MdfA from *E. coli* as a representative MFS efflux pump to investigate factors that can affect its conformational transition in silico. Molecular dynamics (MD) simulations of MdfA’s inward and outward conformations revealed an intermediate state between these two conformations. By comparison of the subtle differences between the intermediate state and the average state, we indicated that conformational transition from outward to inward was initiated by protonation of the periplasmic side. Subsequently, hydrophilic interaction of the periplasmic side with water was promoted and the regional structure of helix 1 was altered to favor this process. As the hydrophobic interaction between MdfA and membrane was also increased, energy was concentrated and stored for the opposite transition. In parallel, salt bridges at the cytoplasmic side were altered to lower probabilities to facilitate the entrance of substrate. In summary, we described the total and local changes during MdfA’s conformational transition, providing insights for the development of potential inhibitors.

## 1. Introduction

Overuse and misuse of antibiotics accelerated the explosion of antibiotic resistance all around the world [1]. Antibiotic treatments of Gram-negative pathogens, such as *Escherichia coli*, *Klebsiella pneumoniae* and *Enterobacter cloacae*, are becoming more and more difficult due to the spreading of antibiotic resistance [2]. Among the factors that can reduce antibiotic susceptibilities of Gram-negative pathogens, efflux pumps play a crucial role by transporting a broad range of substrates out of the cytoplasm [3]. Up to now, six families of proton driven efflux pumps have been identified in Gram-negative pathogens, namely resistance/nodulation/cell division (RND) family, multidrug and toxic compound extrusion (MATE) family, major facilitator superfamily (MFS), small multidrug resistance (SMR) family, proteobacterial antimicrobial compound efflux (PACE) family, and p-aminobenzoyl-glutamate transporter (AbgT) family [4,5,6]. Among these different efflux pumps, MFS family efflux pump is the largest class of secondary transporters and is present in all life kingdoms [7]. In bacteria, various MFS efflux pumps have been reported, such as MdfA from *E. coli*, KmrA from *K. pneumoniae* and SmvA from *Salmonella enterica* [8,9,10]. Different from the tripartite RND family efflux system, MFS efflux pump is a single protein embedded in the inner membrane, while these small efflux pumps have been demonstrated to confer resistance to clinical important antibiotics of quinolones [11,12]. More seriously, plasmid-encoded MFS efflux pumps are discovered from clinical isolates, raising concerns about their horizontal transfer [13]. Therefore, understanding the mechanism of the transport cycle is still important because many questions in this process remain to be addressed.

Efflux pumps of the MFS family share a similar folding topology [14], and the structural features are likely to be the basis for the functions common to most members of the MFS family. Studies show that MFS proteins contain a 12 transmembrane helix core composed of two six-helix rigid domains forming a central transmembrane channel [15]. Based on these structures, a rocker-switch model is proposed for this dynamic process [16]. In this mechanism, MFS efflux pumps are believed to switch between its inward and outward conformations, which represent the accommodating and extruding mode, respectively [17]. From the energetic point of view, the inward-facing conformation is believed to be the excited state and the outward-facing one is the ground state [18]. However, for the structures experimentally characterized or computationally generated from Gram-negative pathogens, most of them are inward-facing conformations (e.g., YajR from *E. coli* and KmrA from *K. pneumoniae*) [19]. As an important method for understanding the transport mechanism, molecular dynamics (MD) simulations suggest that the driving forces of the conformational switch are composed of internal ionic interactions, hydrophobic interaction with membrane, and hydrophilic interaction with water [19]. However, simulations of MFS efflux pump are often restricted by the lack of the corresponding outward-facing structures [20,21]. Therefore, comprehensive simulations of MFS efflux pumps are still essential to describe this dynamic process.

MD simulation and docking have been demonstrated as useful for the development of protein inhibitors. Nevertheless, drug transport, not only for MFS family but also for the other families, is a dynamic process accomplished by large conformational transitions [22,23]. Theoretically, conformational transitions can be observed in sufficient long simulation trajectories. However, within the available computational resources, it is still difficult to observe desired conformational transition via conventional MD [24]. To solve this problem, modified simulation methods have been applied for a large conformational switch, such as targeted MD, steered MD, and high-temperature MD [25,26]. These methods are well-suited for the exploration of phase space and efforts have been made to eliminate the bias [24]. However, conventional molecular dynamics are still believed to be high in accuracy for the description of a protein’s trajectory. Therefore, conventional simulating of both of the conformations of MFS efflux pumps and discovering the intermediate state are anticipated to reveal the factors that favor the conformational transition.

**Figure 1:**
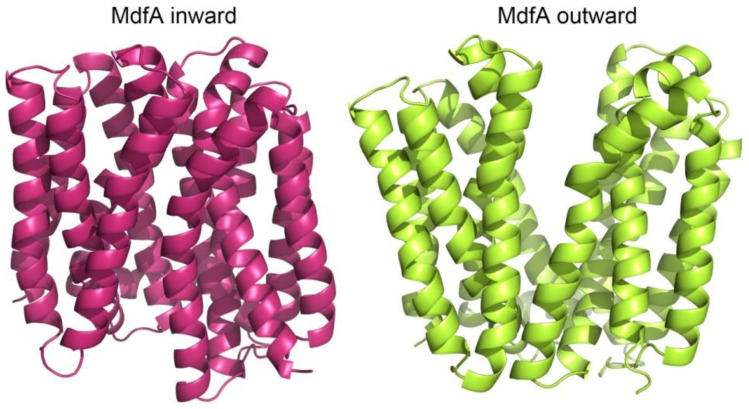
Inward (4zp0) and outward (6gv1) conformations of MdfA. Structures were visualized in Pymol.

After searching the structural data from Enterobacteriaceae pathogens, we found an MFS efflux pump MdfA from *E. coli*, an antiporter that contains both of the characterized inward and outward structures (4zp0 for inward and 6gv1 for outward, swapping R131Q of MdfA inward structure, Figure 1) [27,28,29]. Thus, we aim to analyze the differences between the two structures by comprehensive simulations of them and to find the important factors related to the conformational transitions. An intermediate state was detected between its inward and outward conformations. We analyzed the subtle differences between these structures and revealed the possible factors that facilitate the conformational transitions. In summary, our data provide insights into the mechanism of the conformational transitions of MFS efflux pumps.

## 2. Results

### 2.1. An Intermediate State between Inward and Outward Conformations of MdfA

**Figure 2:**
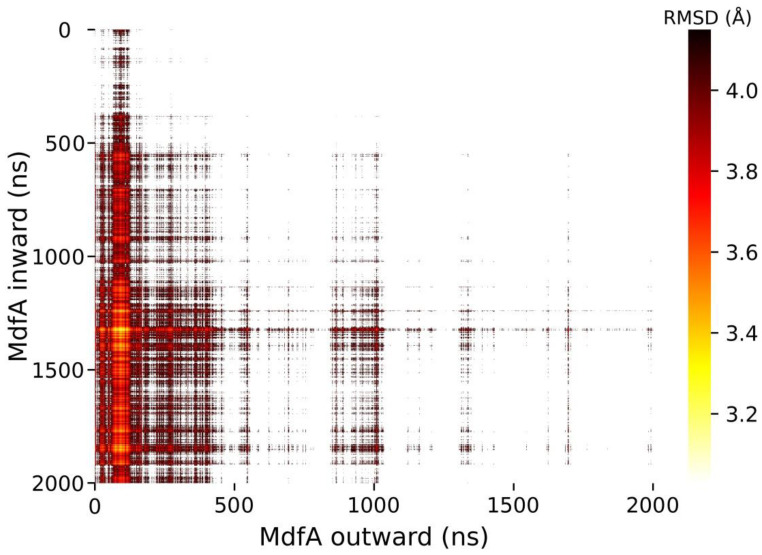
Analyses of MD simulations revealed an intermediate state (snapshot 1325 for inward and 74 for outward) between MdfA^inward^ and MdfA^outward^. For each pair of snapshots, structures were superposed and RMSD was calculated. Heatmap was made in ‘Matplotlib’ module of Python and RMSD values over 4.1 Å were masked.

Since large conformational transition is difficult to observe in conventional MD simulation, we calculated the structural similarities of MdfA^outward^ and MdfA^inward^ during the whole simulations (Figure 2). In general, more than 70% of the snapshots displayed low structural similarities with RMSD values over 4.2 Å. Interestingly, we detected two snapshots from these simulations which showed the lowest RMSD of 3.03 Å. The two snapshots were located at the initial stage of MdfA^outward^ and the over half stage of MdfA^inward^. Since the initial RMSD between MdfA^inward^ and MdfA^outward^ was 4.95 Å, the identified snapshots had nearly 40% decrease in RMSD values, which can be regarded as an intermediate state between the two conformations and can provide detailed information for the conformational transition.

**Figure 3:**
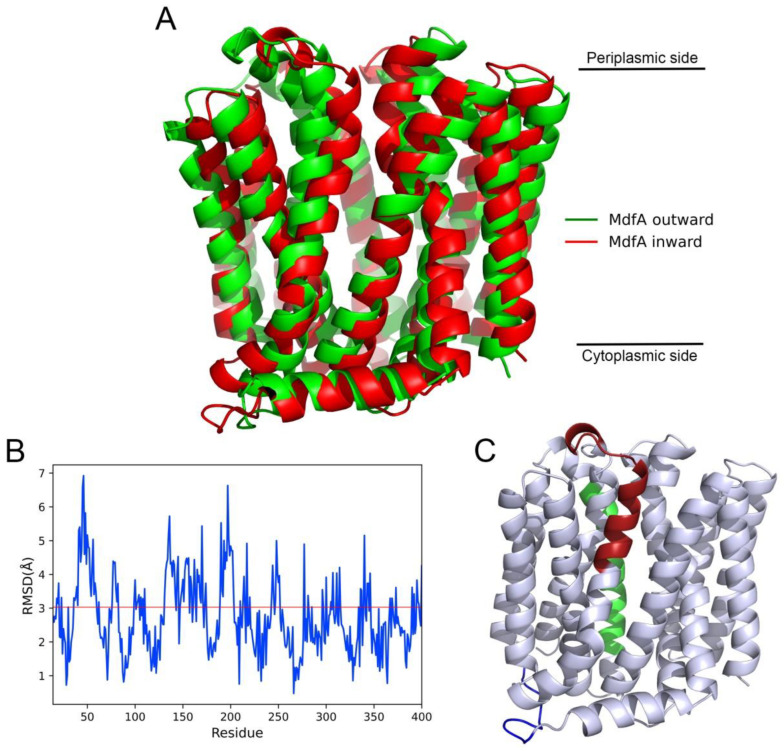
Alignment of MdfA^inward1325^ and MdfA^outward74^ revealed their subtle differences. (**A**): Alignment of superposed MdfA^inward1325^ and MdfA^outward74^. (**B**): RMSD values of each residue between MdfA^inward1325^ and MdfA^outward74^. Red line indicated the average RMSD between MdfA^inward1325^ and MdfA^outward74^. (**C**): Visualization of the regions that had the highest RMSD. Red: Helix 1 (Residue 45–62); Green: Helix 5 (Residue 150–160); Blue: Hinge loop (Residue 195–200).

Next, we visualized the structures (MdfA^inward1325^ and MdfA^outward74^) which had the lowest RMSD value and superposed them into a coordinated system (Figure 3A). MdfA^inward1325^ and MdfA^outward74^ had similar structures in the middle of the central helices, while moderate differences were found at the cytoplasmic and periplasmic side of MdfA. To obtain detailed differences between MdfA^inward1325^ and MdfA^outward74^, we calculated the RMSD value of each amino acid (Figure 3B). The results indicated that the highest RMSD values were concentrated in three areas, namely periplasmic side of helix 2, periplasmic side of helix 5, and the hinge loop connecting N- and C-repeats (Figure 3C, labeled with red, green and blue, respectively) [30]. Surprisingly, these major differences were all located in the N-repeat of MdfA between MdfA^inward1325^ and MdfA^outward74^, which suggested that movement of N-repeat was important for the conformational transitions, and a crucial step for MdfA’s conformational transition was the structural alterations at the top area of the N-repeat.

### 2.2. Flexibilities and pK_a_s of MdfA

**Figure 4:**
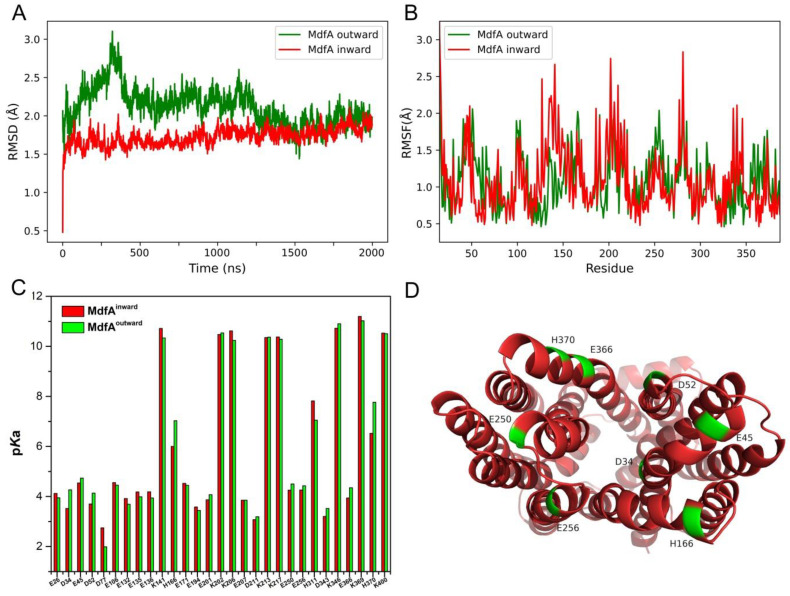
Flexibilities and p*K*_a_ values of MdfA. (**A**): Backbone RMSD of MdfA^inward^ and MdfA^outward^. (**B**): RMSFs of MdfA^inward^ and MdfA^outward^. (**C**): p*K*_a_ values of each residue of MdfA^inward^ and MdfA^outward^. (**D**): Periplasmic view of the residues that had higher p*K*_a_ values at outward-facing conformation.

The intermediate state of MdfA occurred only once during the MD simulations. To explore more factors that could contribute or restrict MdfA’s conformational transitions, we calculated the flexibilities of MdfA^inward^ and MdfA^outward^ (Figure 4A,B). The backbone RMSD of MdfA^inward^ reached a stable value around 1.7 Å from 5 ns to the end of the simulation. In contrast, RMSD of MdfA^outward^ was promoted to 3.0 Å in the initial stage, followed with gradually decreased values to around 2.0 Å. In the final 500 ns of simulation, there was no significant difference in flexibility between MdfA^inward^ and MdfA^outward^.

In parallel, amino acid flexibilities were calculated (Figure 4B). MdfA^inward^ displayed significantly increased RMSF values in several regions, namely helix 5 (Residue 130–150), hinge loop connecting N- and C-repeats (around residue 200), loop connecting helix 8 and helix 9 (around residue 280), and loop connecting helix 10 and helix 11 (residue 340–345). Interestingly, these three loops were all located at the cytoplasmic side of MdfA. In contrast, there were three regions of MdfA^outward^ that have moderately increased RMSFs, namely helix 2 (residue 55–60), loop connecting helix 9 and 10 (residue around 250) and helix 11 (residue 350–360), and these loops were located at the periplasmic side of MdfA. Collectively, helices near the surface between N- and C-repeats had varied flexibilities. Moreover, these loops at each side may have underestimated roles in MdfA’s conformational transitions.

At the same time, the p*K*_a_ value of each residue in MdfA^inward^ and MdfA^outward^ was calculated (Figure 4C). As expected, higher p*K*_a_ values were detected at the mouth of the relative opening side, while the p*K*_a_ values of residues in the central helices were kept unchanged between MdfA^inward^ and MdfA^outward^. Residues with higher p*K*_a_ values of MdfA^outward^ were visualized (Figure 4D) and these residues were located around the opening of MdfA’s periplasmic side, providing higher possibilities for protonation at the ground state. However, p*K*_a_ values of the residues in each simulation were not changed during these simulations, and the reason was deduced because of the rarely altered conformations.

### 2.3. MdfA^inward^ and MdfA^outward^ Had Altered Interactions with Water and Membrane

**Figure 5:**
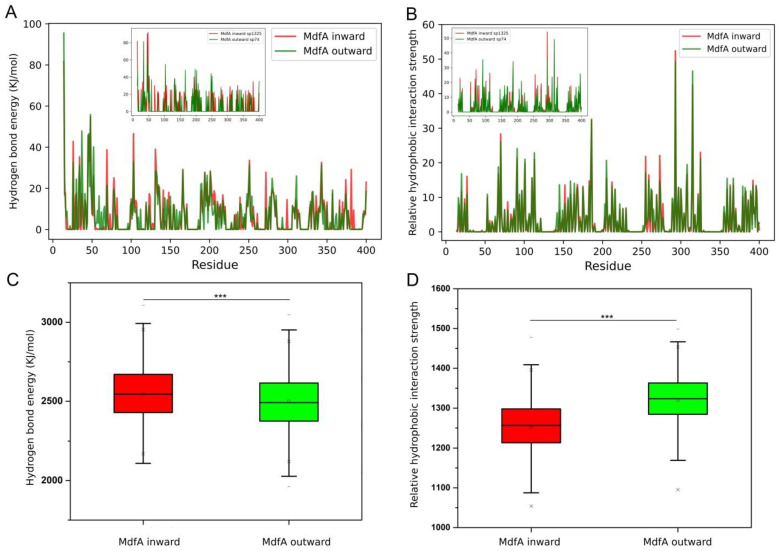
MdfA had different interaction strengths with water and membrane. Local changes of interactions with water (**A**) and membrane (**B**), and the embedded pictures indicated the transient interaction strengths of MdfA^inward1325^ and MdfA^outward74^. Global changes of interaction between MdfA^inward^ and MdfA^outward^ (**C**,**D**). *** represented the significance (*p* < 0.001).

The driving force of the conformational transition of MFS efflux pumps are composed of several factors, and its hydrophilic interaction with water and hydrophobic interaction with membranes are two important parts, due to the fluidity of water and membrane [17]. Therefore, we calculated these average interaction strengths and the transient strengths at the intermediate state (Figure 5). In general, MdfA^inward^ had stronger interaction with water for most of the residues. However, at the intermediate state, hydrophilic interactions of MdfA^inward^ were lower than those of MdfA^outward^ at several loop regions (Figure 5A). Conversely, significantly increased hydrophilic interaction strengths were observed for MdfA^inward1325^ at the loop near A49, the area of which also displayed the lowest similarities between MdfA^inward1325^ and MdfA^outward74^ (Figure 3B).

In parallel, we also calculated the average and transient hydrophobic interaction strengths with membrane (Figure 5B). The results indicated that there was no significant difference on average interaction strength between MdfA^inward^ and MdfA^outward^. However, much stronger interactions of MdfA^inward1325^ were observed compared to MdfA^outward74^ in helix 2 (residue 60–70), helix 8 (residue 255–265), and helix 9 (residue 285–290). These differences indicated that even though the inward conformation was regarded as excited mode, MdfA’s transition from inward back to outward may be favored by the regional promoted hydrophobic interaction with membrane. These elevated interactions may act as storage of energy for the transition back to the ground state.

### 2.4. Reduced Ionic Interactions at the Cytoplasmic Side Were Crucial for MdfA’s Activation

**Figure 6:**
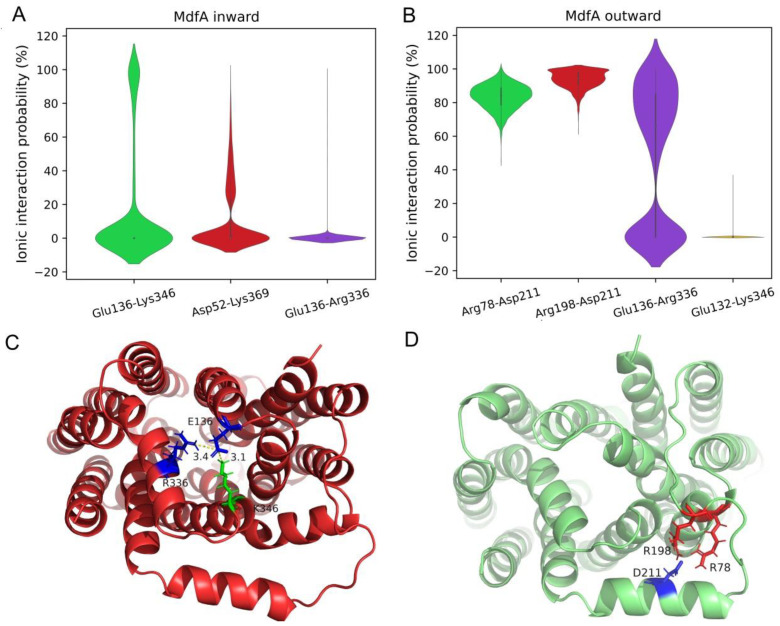
Salt bridges were altered between MdfA^inward^ and MdfA^outward^. (**A**,**B**): Violin plot of possibilities of salt bridges between N- and C-repeats. (**C**,**D**): Visualization of salt bridges of MdfA^inward1325^ and MdfA^outward74^ at the cytoplasmic side.

Previous studies demonstrate that ionic interactions provide another important driving force for the conformational transition of a MFS efflux pump [19,31,32]. Therefore, possibilities of the salt bridges between N- and C-repeats were calculated (Figure 6). In general, much stronger ionic interactions were detected in MdfA^outward^ than in MdfA^inward^. For MdfA^inward^, three salt bridges were detected during the whole simulation with low possibilities, and two of them were found at the cytoplasmic side (Glu136-Lys346 and Glu136-Arg336). At the periplasmic side, Aps52-Lys369 also had low level of interaction strength. However, for the intermediate state of MdfA^inward1325^, stronger ionic interactions were observed between Glu136-Lys346 and Glu136-Arg336 (Figure 6C), showing significant differences from the average situation. These salt bridges were detected across the mouth of the substrate entrance, suggesting that these salt bridges on the cytoplasmic side may provide important forces for the transition back to the ground mode even though this process was more or less automatic. At the same time, four salt bridges were detected at the cytoplasmic side of MdfA^outward^ with much higher interaction possibilities (Figure 6D). More importantly, these salt bridges had altered positions compared to those in MdfA^inward^. Therefore, we can deduce that for MdfA’s activation, elimination of these strong ionic interactions at the cytoplasmic side might be a prerequisite.

### 2.5. MdfA^inward^ Had Secondary Structure Changes in the Central Helix

**Figure 7:**
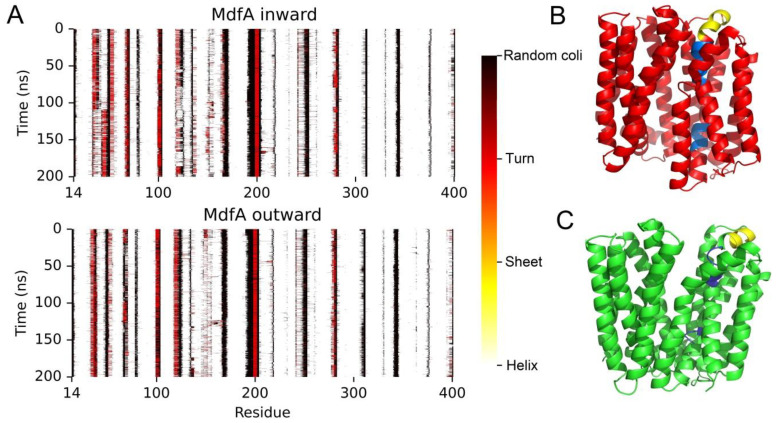
MdfA^inward^ had altered secondary structure of helix 1. (**A**): Heatmap of the secondary structures of MdfA^inward^ and MdfA^outward^ throughout the two simulations. (**B**): Loops in helix 1 were changed into helix (Blue) for MdfA^inward1325^, and the top of helix 1 (Yellow) became upright compared to the structures in MdfA^outward^ (**C**).

To obtain more details that can affect MdfA’s conformational transition, we calculated the secondary structures of MdfA^inward^ and MdfA^outward^ during the whole simulations (Figure 7). As a result, we discovered a special conformational change in the central helix 1 (residue 32–35). In the initial structures of both MdfA^inward^ and MdfA^outward^, there exist two loops inside the central helix 1. For MdfA^outward^, these loops were not affected throughout the simulation (Figure 7C). On the contrary, these loops of MdfA^inward^ were changed into helices in the second half of simulation, and the helices were kept stable to the end (Figure 7B). These data indicated that helix 1 was changed into a rigid mode, and this was in accordance with the flexibilities of residues in this region (Figure 4A,B). Helix 1 was located in the center of MdfA and had a close distance with helix 7 of C-repeat. Coincidently, the intermediate state of MdfA was obtained at 1325 ns of MdfA^inward^ and 74 ns of MdfA^outward^ (Figure 2), and at this time point, there is no loop structure in the helix 1 of MdfA^inward^ (Figure 3A). Therefore, switching from loop to helix of these regions might be another crucial step for the activation of MdfA.

## 3. Discussion

Efflux pumps play important roles in bacterial metabolism. Nevertheless, efflux pumps usually confer antibiotic resistance and cause the failure of antibiotic therapy [33]. Drug transport by these efflux pumps is accomplished by their large conformational transition. However, in silico analysis of these efflux pumps are restricted by the limited computational power, which is unable to obtain large conformational transition in a confined simulation process [34]. Even though modified simulations have been created to reduce the need of computational resources, the reduction of accuracy will conceal important details that can affect the conformational transition, especially for a simulation with membrane. At the same time, for MFS efflux pump structures captured in the wet-experiments, inward conformations occupy most of them. Therefore, computational analyses of MFS efflux pumps are also confined by the lack of outward conformations [17,19]. Here we employed conventional MD simulations based on the well-characterized structures of MdfA and discovered an intermediate state between its inward and outward conformations. By comparing structural variations with the intermediate state, important details that affect its conformational transitions can be provided to intensively understand the drug export process.

### 3.1. Two States of MdfA

The conventional rocker-switch model is commonly used for the transport mechanism of a MFS efflux pump [30]. Based on the energetic point of view, the outward and inward conformations are believed to be the ground state and the excited state, respectively [18]. In such a mechanism, these two conformations differ by a nearly 35° rotation of one domain relative to another. However, recent biochemical studies of MdfA suggest that there may exist a special occluded conformation with ligand included. By modifying MdfA and measuring the distances with Double Electron Electron Resonance, Yadeni et al. demonstrate that MdfA is a relative flexible MFS efflux pump in membrane [35]. Notably, the occluded state of MdfA displays both a closed periplasmic and cytoplasmic side. Moreover, a substrate-responsive lateral gate is identified which is open toward the inner leaflet of membrane but closes upon drug binding. Therefore, the mechanism for MdfA or the other MFS efflux pumps may still under debate. The differences are deduced due to the absence of ligands in our simulation. Herein, according to the RMSD values of the intermediate state (Figure 3B), the major differences are located in the top area of MdfA’s N-repeat, while much less differences are found in the C-repeats. This suggests that MdfA’s conformational transition is largely based on the movement of N-repeat. At outward-facing conformation, several important residues at the periplasmic side (e.g., Asp 34, Glu 45, Glu 256) have higher p*K*_a_ values ready for protonation, and these residues have been demonstrated as crucial for the activity of MdfA [27]. On the other hand, higher p*K*_a_ values are detected for residues at the cytoplasmic side of MdfA^inward^ (e.g., Glu 132, Glu 135, Glu 136, Lys 141, Lys 217 and Lys 206), which means MdfA is ready to accommodate substrate at an inward-facing conformation. Except for the residues at each side of MdfA, Glu 26, which locates in the center of a drug binding pocket, also display slightly higher p*K*_a_ value at the inward-facing conformation (4.12 for inward and 3.94 for outward). Glu 26 has also been demonstrated as crucial for MdfA’s activity in previous research [27], and its elevated p*K*_a_ value suggests an efficient process for substrate entrance and binding.

The other driving forces for MdfA’s conformational transition include internal salt bridge and the interaction with water and membrane, which we have proved important for the action of MdfA’s ortholog KmrA in *K. pneumoniae* [19]. Here we divide the conformational transition process into two parts to address the corresponding driving forces.

### 3.2. Transition from Outward to Inward

The outward conformation is believed to be the ground state of MdfA, and the most important difference with MdfA^inward^ is the stronger interactions of salt bridges at the cytoplasmic side (Figure 6B), which displayed significantly higher possibilities throughout the simulation. These interactions might lay the foundation for the stability of MdfA at the ground state [18,36]. The protonation of Asp 34 at the periplasmic side is then deduced to partially eliminate the ionic interactions, and cause local conformational change of helix 1 [37]. The loop adjacent to Asp 34 is transitioned to helix, increasing the rigidity of helix 1 and forcing the periplasmic side of helix 1 (Figure 7B, colored with yellow) become upright. This change might favor the overall movement of the N-repeat. By transporting one proton across membrane, membrane potential can also provide extra energy, which produces torques that rotate the two repeats oppositely for conformational change [37,38]. With the transition from outward to the intermediate state, residues at the periplasmic side (Gln 103, His 166, Glu 250 and Gly 251) have increased hydrogen bond energies with water (Figure 5A). Moreover, two important residues at the cytoplasmic side Thr 197 and Glu 201 also have increased interactions with water. Interestingly, the two residues are located adjacent to the strong salt bridges at the cytoplasmic side (Figure 6B). Thus, the enhanced interactions with water of this region are deduced to affect the ionic interactions between N- and C-repeats, re-allocating these ionic interactions to the inward-facing mode. Meanwhile, the overall change of hydrophobicity confers redistribution of MdfA’s hydrophobic interaction with membrane (Figure 5B). Stronger hydrophobic interactions with membrane are concentrated in some residues at the cytoplasmic side (e.g., Leu 20, Trp 70, Leu 273, Leu 274 and Trp 293). Notably, Leu 20 and Trp 70 are located in the conserved region of motif D and motif A across all the MFS efflux pumps [38], meaning they may have a universal role in forcing the conformational transitions. At the intermediate state of MdfA^inward^, these interactions are even stronger. Therefore, these interactions could be classed into energy storage for the transition back to the ground state. Taken together, we hypothesize that protonation initiates the conformational transition, which then leads to the structural changes in helix 1 and the increased interactions with water at both sides. As ionic interactions at cytoplasmic side are altered, inward-facing conformation is transitioned and hydrophobic interactions with membrane are concentrated.

### 3.3. Transition from Inward to Outward

From an energetic point of view, transition from inward to outward is more or less automatic. The contributing factor of this process is deduced as deprotonation, which occurs in the inward facing conformation due to the alkaline condition of the cytosol and the combination with substrates [39]. Notably, salt bridges are in low possibilities during the simulation of inward conformation, but stronger ionic interactions were detected at the intermediate state MdfA^inward1325^ (Figure 6B). This suggests that salt bridges still play a crucial role in the conformational transition from inward to outward, and we deduce that this process can be triggered by MdfA’s combination with substrates [40]. With promoted interactions with membrane at some regions, conformational transition is initiated back to the ground state with a mechanism similar to the first outward-to-inward transition. With the release of substrate to the periplasm, hydrophobicity and membrane potential are changed back to the ground state.

In summary, we propose a mechanism of MdfA’s conformational transition based on the intermediate state between its inward and outward conformations. Through comparing different interactions with the intermediate state, we demonstrate the factors that drive the transition and reveal the detailed changes of regional structures. However, there are still mechanisms unanswered. Even though the conformational changes of MdfA^inward^ and MdfA^outward^ have been investigated, these data might be slightly different from the simulations that contain substrates. Moreover, various studies have proved that MdfA can confer multidrug resistance such as ciprofloxacin and rifampicin [41,42]. However, these antibiotics belong to different families, and they may interact with MdfA at distinct residues. Therefore, more efforts are essential to simulate the process that contains substrates’ entrance and release.

## 4. Materials and Methods

### 4.1. Structures and Software

The inward (4zp0) and outward (6gv1) structures of MdfA were downloaded from RCS PDB bank followed by deleting ligands. After the alignment of the amino acid sequences, we found that there existed one residue substitution (Gln 131 for outward and Arg 131 for inward). To make the simulations comparable, we made the swap R131Q of MdfA inward structure followed by energy minimization. Then the two structures were applied as the initial states of the two simulations. Structural modification and molecular dynamics simulations were conducted in the commercial YASARA Structure software suit (Version 21.6.17, YASARA Biosciences GmbH, Vienna, Austria) since we have the license.

### 4.2. Molecular Dynamics

All the MD simulations were carried out in YASARA Structure using Amber 14 force field with periodic boundary condition. The standard marco ‘md_run_membrane’ was applied for all the simulations. First, PDB file of MdfA was cleaned and the ligand was removed. The simulation was set up automatically by first scanning the protein for exposed transmembrane helices. The major axis vectors of these helices were summed up to obtain the major axis of the protein, which was then oriented along the Y-axis, normally with respect to the plane of the membrane and the XZ-plane. The best shift of the membrane along this major axis was obtained by scanning the protein for the region with the largest number of exposed hydrophobic residues. Having placed an equilibrated membrane structure, the system was enclosed in a simulation cell of size [90 × 90 × 90] Å. The protein was temporarily scaled by 0.9 along the XZ-axes, and strongly clashing membrane lipids were deleted. The temporary protein scaling, which was needed to avoid the deletion of too many lipids around the protein, was then slowly removed during a short simulation at 298 K in vacuo. The force field was AMBER14 with Lipid17 parameters for non-standard residues. As soon as the protein had reached its original size again, the protein side-chain p*K*as were predicted. Protonation state was assigned according to pH 7.4, and the simulation cell was filled with 34,133 water molecules, 0.9% NaCl and counter ions. After energy minimization, the main simulation was then run with PME and 8.0 Å cutoff for non-bonded real space forces, a 4 fs time-step, constrained hydrogen atoms, and at constant pressure and temperature (NPT ensemble). During the initial 250 picoseconds, the membrane was restrained to avoid distortions while the simulation cell adapted to the pressure exerted by the membrane. Each simulation was performed for 2000 ns at 310 K and 1 bar with two replicates. The snapshot was recorded every 0.1 ns. The marco used in MD simulation are available from http://www.yasara.org/md_runmembrane.mcr (Accessed on 6 January 2012).

### 4.3. Data Analysis

Analysis of the trajectory of simulation was also conducted in YASARA. Root Mean Square Deviation (RMSD) and Root Mean Square Fluctuation (RMSF) were used to evaluate the total and local flexibilities. Ionic interaction (salt bridge) was identified if the distance between positively and negatively charged residues was lower than 5 Å. Relative hydrophobic interaction strength defined by YASARA Structure was used to determine the hydrophobic interaction between MdfA and membrane. Briefly, relative hydrophobic interaction strength between two hydrophobic groups ranged from 0 to 1 based on their distances, and the interaction strength between residues was the total strength of each hydrophobic group. Similarities between inward and outward conformations were calculated during the simulations. For each alignment, structures from the two simulations were superposed by residue name to make them in the same coordination. Then RMSD between the two structures were calculated. Since the snapshot was recorded each 0.1 ns and the simulations were conducted for 2000 ns, a 2000 × 2000 matrix was filled and a heatmap was plot. All the plots were made in Python ‘Matplotlib’ module and the structures were visualized in Pymol.

## 5. Conclusions

MdfA from *E. coli* is a representative MFS efflux pump and can confer multidrug resistance. By using the outward and inward conformations of MdfA, we investigated the details that related to its conformational transitions. We identified an intermediate state of MdfA and made comparisons on flexibility, hydrophobicity, internal interactions, and secondary structures. Our data proposed a mechanism for MdfA’s conformational transitions and provided insights for developing new inhibitors of MFS efflux pump.

## Data Availability

Research data are available from email request to the corresponding author.
